# Precision residential greenspace, urban environmental exposures, and incident dementia among older adults in Sweden: A 16‐year cohort study

**DOI:** 10.1002/alz.71835

**Published:** 2026-09-28

**Authors:** Cecilia U. D. Stenfors, Jing Wu, Giulia Grande, Andrei Pyko, Mare Löhmus, Göran Pershagen, Debora Rizzuto

**Affiliations:** ^1^ Department of Psychology Stockholm University Stockholm Sweden; ^2^ Aging Research Center Department of Neurobiology Care Sciences and Society Karolinska Institute Stockholm Sweden; ^3^ Institute of Environmental Medicine Karolinska Institute Stockholm Sweden; ^4^ Center for Occupational and Environmental Medicine Karolinska Institute Stockholm Sweden

**Keywords:** Alzheimer's disease, cognitive aging and resilience, nature‐based solutions, residential environmental exposures, urban planning

## Abstract

**INTRODUCTION:**

Residential greenspace is linked to mental and cardiometabolic health, but longitudinal studies on detailed greenspace and dementia incidence are limited.

**METHODS:**

We followed 2993 dementia‐free participants in the Swedish National Study on Aging and Care Kungsholmen for 16 years; 477 developed clinically diagnosed dementia. Greenspace was assessed using normalized difference vegetation index within 100‐, 250‐, and 500‐m residential buffers. Cox models estimated hazard ratios (HRs) per interquartile‐range greenspace increment and tested effect modification by air pollution, road traffic noise, blue space, age, sex, and apolipoprotein E‐ε4 (APOE‐ε4).

**RESULTS:**

Higher greenspace within 100 m was associated with lower dementia incidence (HR = 0.79, 95% confidence interval [CI] = 0.64–0.96), whereas associations for larger buffers were attenuated. Associations were stronger in women (HR = 0.69, 95% CI = 0.54–0.89) than men (HR = 0.93, 95% CI = 0.71–1.24).

**DISCUSSION:**

Close residential greenspace may reduce dementia risk in older adults, particularly women.

## Highlights

- Longitudinal study of an urban aging cohort with 16 years of follow‐up
- Detailed longitudinal assessment of residential exposures and individual factors
- Higher residential greenspace within 100 m associated with lower dementia incidence
- Moderation by traffic noise, air pollution, blue space, age, sex, and apolipoprotein E‐ε4 (APOE‐ε4) was tested
- Protective greenspace effects were stronger among women than men

## 1 BACKGROUND

Adequate cognitive function is vital in daily functioning, and cognitive impairment and dementia in the elderly population are major public health concerns with vast individual and societal costs.^1^ In 2015, about 47 million people worldwide were living with dementia, and the projected increase is 66 million by 2030 and 115 million by 2050.^2^ In Sweden alone, the projected increase is from ca 150,000 today to 250,000 cases in 2050.^3^ Identifying and targeting modifiable protective‐ and risk factors is thus vital.

Importantly, research suggests that qualities in the living environment in terms of natural spaces, notably greenspace (vegetation), are associated with better mental health outcomes, including better sleep^4^ and lower depression, stress,^5^, ^6^, ^7^ and cardiometabolic health outcomes.^5^, ^8^, ^9^ These health domains are important determinants for cognitive preservation versus decline and dementia.^2^, ^10^, ^11^ Recently, research findings have also emerged which indicate that residential greenspace may be linked to better cognitive functioning and lower risk of dementia.^12^, ^13^, ^14^, ^15^ However, results vary across studies of greenspace and dementia, and methods have mostly been constrained to more crude area‐level assessments of residential greenspace, at the neighborhood or broader level scales, rather than assessing greenspace in the immediate surrounding of individuals’ addresses.^12^, ^13^, ^14^, ^15^ This increases the risk of exposure misclassification and may contribute to the variable findings in prior studies.^5^

Residential greenspace may contribute to increased cognitive health and resilience via several pathways, broadly including (1) psychophysiological restorative effects, (2) health behaviors, and (3) buffering against environmental hazards.^9^, ^12^ Firstly, observed restorative psychophysiological effects of greenspace exposure include improved affect,^16^ cognitive performance,^17^ and lowered mental and physiological stress^18^, ^19^ in experimental studies. Benefits of residential greenspace in observational epidemiological studies include better sleep quality^4^ and lower depressive symptoms and other depression indicators, mental stress,^6^, ^7^ and physiological stress biomarkers.^20^ Greenspace in the immediate residential surroundings seems to be especially important to mental health outcomes, as individuals are exposed to this greenspace on a daily basis while in the residential environment, as suggested in recent population‐based studies using detailed greenspace measurements of the individual residential environment.^4^, ^6^, ^7^ Secondly, residential greenspace can facilitate health‐supporting behaviors, including physical activity^21^ and social connection,^22^ that are linked to better mental health and reduced risk of cardiovascular disease (CVD), cognitive decline, and dementia.^2^ Thirdly, greenspace may buffer against environmental hazards, including traffic noise and air pollution^23^, ^24^ that can have adverse effects on health and cognition, for example via inflammatory, atherosclerotic, and stress‐related processes.^25^, ^26^, ^27^ These environmental hazards can also covary with and confound greenspace associations with health outcomes. Furthermore, “blue spaces” (i.e., open water bodies like lakes, seas, and rivers) are also regarded as restorative natural environments that can provide some similar benefits as greenspace and have been associated with psychological restorative processes and higher physical activity,^28^ which in turn may help support the preservation of brain health and cognitive functioning as described above. Thus, also considering blue space access can further our understanding of whether the role of residential greenspace for dementia also depends on access to blue space.

However, studies are yet limited on the role of detailed individual‐level residential greenspace for the development of dementia that is carefully assessed and followed up longitudinally, while also considering other environmental and individual risk factors.^12^, ^14^, ^15^ The aims of the present study were thus to investigate the role of individual residential greenspace and greenspace closeness around the home, for dementia incidence in a Swedish longitudinal aging cohort, in an urban area of the city of Stockholm, and the potential modifying effects of age, gender, residential blue‐space access, air pollution and road traffic noise, as well as genetic risk profile for dementia in terms of being a non‐carrier or carrier of the apolipoprotein E‐ε4 (*APOE* ε4) allele conferring increased risk of Alzheimer's disease (AD).^2^

## 2 METHODS

### 2.1 Study population and data collection

The current study is based on data collected in the Swedish National study on Aging and Care in Kungsholmen (SNAC‐K), which is an ongoing population‐based cohort from the Kungsholmen district, Stockholm, Sweden. At baseline (2001–2004), 3363 out of 4590 alive and eligible persons aged ≥60 years (73,3% participation rate) participated and have been followed up regularly over 16 years: every 6 years for the young‐old cohorts (aged < 78 years) and every 3 years for the older cohorts (aged ≥78 years). Participants underwent extensive clinical examinations, interviews, and assessments by trained physicians, nurses, and psychologists. Medication use from the inpatient register was classified according to the World Health Organization Anatomical Therapeutic Chemical (ATC) classification system. The history of participants’ residential addresses (between 1990 and 2018) was obtained via The Swedish Tax Agency. Through linkage to the individual residential address coordinates, annual assessments were made of residential greenspace, blue space, traffic noise, and air pollution.

For the present study, participants with a diagnosis at baseline of dementia (*n* = 240), intellectual disability (*n* = 1), or schizophrenia (*n* = 16) or with a Mini‐Mental State Examination (MMSE) score < 24 (*n* = 103), were excluded. In addition, participants with missing dementia information at baseline (*n* = 10) were excluded, leaving 2993 participants without dementia at baseline in the analytical study sample.

Ethical approval was obtained from the Karolinska Ethics Committee (Stockholm, Sweden) and Regional Ethical Review Board in Stockholm for all phases of SNAC‐K.

### 2.2 Assessment of dementia

The diagnosis of all‐cause dementia was made in accordance with the Diagnostic and Statistical Manual of Mental Disorders, 4th Edition Text Revision (DSM‐IV‐TR) and follows a three‐step procedure. The first diagnosis is made by the examining physician and reviewed by a second physician. In case of disagreement in the diagnosis, a neurologist who was external to the data collection provided the final diagnosis. For those participants who died between follow‐up assessments in SNAC‐K (and who did not have a dementia diagnosis at the last follow‐up), we supplemented the diagnosis of dementia by reviewing the Swedish National Cause of Death Register and clinical medical records.

### 2.3 Assessment of residential greenspace

Residential greenspace was assessed for the participants’ residential address histories by using the satellite‐based Normalized Difference Vegetation Index (NDVI).^29^ Satellite images from Landsat 5 TM, Landsat 7, and Landsat 8 (during the summer months, May 1 to September 30) were used to derive the annual max‐values of NDVI, with 25 × 25 m resolution. The NDVI is a measure of green vegetation foliage within a given zone and is estimated from visible red (RED) and near‐infrared (NIR) radiation interacting with photosynthetic tissue in plants, using the following calculation formula:

$$NDVI=\frac{NIR-RED}{NIR+RED}$$

NDVI‐values range from ‐1 to +1, where higher (positive) numbers indicate more green vegetation foliage. The possible impact of cloud contamination (giving rise to “false low values”) was corrected by replacing the annual NDVI values of each 25 m × 25 m pixel with an average value for 5 consecutive years centered at the actual year (i.e., the average of 2 preceding years, the actual year, and 2 following years). Furthermore, since areas covered with blue space (i.e., surface water like lakes, ocean, rivers, etc.) are represented by negative NDVI values, all surfaces covered with water were excluded from the buffer zones to avoid underestimation of greenness NDVI values. To assess individual‐level residential greenspace, annual NDVI values were estimated within 100 m, 250 m, and 500 m Euclidean circular buffer zones (radiuses) surrounding participants’ individual residential addresses from 1990 to 2018, by using QGIS (v. 3.22 and v. 3.24). Then average NDVI values were computed for individual exposure windows during the 5‐year period and the 5‐ to 10‐year period before the event of interest (i.e., before disease onset or end of follow‐up).

**RESEARCH IN CONTEXT**

1. **Systematic review**: The authors reviewed previous studies on residential greenspace, cognitive aging and dementia using conventional sources like PubMed. Prior findings have been mixed. Key limitations include the use of aggregated, area‐level greenspace measures and lacking assessment of the immediate individual residential surroundings, frequent use of register‐based dementia ascertainment, and limited consideration of other environmental and individual risk factors.
2. **Interpretation**: In this 16‐year cohort of 2993 older adults, higher residential greenspace within a 100 m buffer of the home was associated with lower dementia hazard, while controling for demographic, genetic, and environmental factors, including air pollution and road traffic noise. Associations were weaker for larger buffers, stronger among women, and more pronounced among apolipoprotein E‐ε4 (APOE‐ε4) non‐carriers.
3. **Future directions**: Future studies should investigate longitudinal links between individual‐level greenspace exposure, cognitive decline, dementia, and neurodegenerative biomarkers, clarify underlying pathways, and examine these associations in more diverse socioeconomic and urban contexts.

### 2.4 Assessment of covariates

Potential confounding factors and effect modifiers assessed at baseline include age, sex, educational level (elementary, high school, or university and above), individual socioeconomic status (SES) derived from the highest‐held occupation (blue‐collar, white‐collar workers, or entrepreneurs), civil status (married, widow, unmarried, divorced, and partnered), smoking status (never, former, and current smoking), physical inactive defined as those who were involved less than once a week in light and/or intensive activities, alcohol consumption (no or occasional, light to moderate [men: 1–14 drinks per week; women: 1–7 drinks per week], heavy drinking [men: > 14 drinks per week; women: > 7 drinks per week]), body mass index ([BMI; kg/m^2^] calculated as weight [kg] divided by the square of height [meter]), and early retirement defined as the retirement before age 65 years. The information on neighborhood household average income by an average of 1000 to 2000 inhabitants was obtained from Statistics Sweden (https://www.scb.se). Peripheral blood samples were collected and genotyped for the apolipoprotein E (*APOE*) alleles. Participants were categorized as ε4 carriers or ε4 non‐carriers.

Road traffic noise levels at the most exposed façade were assessed based on the methodology in the Nordic studies on occupational and traffic noise in relation to disease (NordSOUND) project.^30^ The day–evening–night sound pressure levels (L_den_) of road traffic noise were calculated. The 5‐year time‐weighted average level of traffic noise before the event, on the sound energy scale, was dichotomized into high and low exposure based on the average level of exposure in the sample (57.9 dB). Yearly levels of PM_2.5_ from 1990 to 2004 were obtained from Gaussian dispersion models with a quadtree receptor grid from 35 to 500 meters. The 5‐year average levels of PM_2.5_ before baseline were calculated to consider the effect of air pollution and then dichotomized into high (≥8.4 µg/m^3^) and low (< 8.4 µg/m^3^) based on the study sample mean. Water coverage at the 100‐m buffer was assessed by Geographical Information Systems software QGIS and land use data from the Swedish Mapping, Cadastral, and Land registration authority. The 5‐year average level of water coverage (blue space access) before the event was calculated and dichotomized into any (> 0) and none (= 0).

### 2.5 Statistical analyses

#### 2.5.1 Main analyses

The association between greenspace and dementia risk was assessed using Cox proportional hazard models by estimating hazard ratios (HRs) and 95% confidence intervals (CIs). The proportional hazard assumption was tested by Schoenfeld residuals. The under‐risk period was from baseline to the date of dementia diagnosis, death, drop‐out, or the end of follow‐up, whichever occurred first. The models were adjusted for the aforementioned covariates in an additive manner.

The effect modification of other environmental factors (air pollution, blue space access/water coverage, traffic noise pollution), age group, sex, and *APOE*‐ ε4 was tested by introducing multiplicative interaction terms in the models. According to the results of these analyses, a 5‐year mean NDVI at 100 m buffer (before the event) was used as the main exposure window for the stratified analyses. In order to relax the type I error, *p‐*values ≤ 0.10 were considered indicative of a significant interaction.

#### 2.5.2 Sensitivity analyses

We compared the characteristics of participants who dropped out due to refusal or moved during follow‐up with those who did not. Because of the data availability, we could only consider air pollution levels before the baseline. Though the PM_2.5_ air pollution levels were stable over the follow‐up period,^31^, ^32^ the misalignments of the environmental exposure periods may be a concern. Thus, we repeated the analyses excluding those participants who moved during follow‐up (*n* = 349) to test the robustness of the main results. To consider the potential selection bias due to dropout, we generated the inverse probability weight (IPW) with logistic regression considering age, sex, and education levels. We re‐ran the analyses and assigned the IPW to each participant that remained in the model.

All analyses were performed with Stata, version 17.0 (StataCorp, TX, USA). The results are reported following the STrengthening the Reporting of OBservational studies in Epidemiology (STROBE) Recommendations.

## 3 RESULTS

### 3.1 Main results

Over the 16‐year follow‐up (mean: 9.7 years) among the 2993 total participants, 477 (16%) were diagnosed with dementia. People exposed to low levels of greenspace tended to be older, have lower SES status, have lower education level, be more physically inactive, subscripted to more medications, and exposed to higher levels of air pollution and road traffic noise compared to those exposed to high greenspace (Table 1). A total of 499 participants dropped out during follow‐ups (Table S1). Those participants were older, had lower education levels and were more physically inactive than those who did not drop out, but the two groups were similar with regards to the residential environment exposures.

**TABLE 1:** Baseline characteristics of the overall sample and by levels of 5‐year average greenspace exposure at 100 m buffer before event.

	Total	Low greenspace	High greenspace	No dementia	Incident dementia
Parameter	*N* = 2,993	*N *= 1,564	*N *= 1,429	*N* = 2,516	*N* = 477
Age at baseline, mean ± SD	73.1 ± 10.4	73.5 ± 10.5	72.7 ± 10.3	71.8 ± 10.3	79.9 ± 8.0
Sex, n (%)					
Men	1,118 (37.4%)	585 (37.4%)	533 (37.3%)	968 (38.5%)	150 (31.4%)
Women	1,875 (62.6%)	979 (62.6%)	896 (62.7%)	1,548 (61.5%)	327 (68.6%)
SES status, n (%)					
Blue collar workers	650 (21.7%)	350 (22.4%)	300 (21.0%)	520 (20.7%)	130 (27.3%)
White collar workers	2,021 (67.5%)	1,042 (66.6%)	979 (68.5%)	1,711 (68.0%)	310 (65.0%)
Entrepreneurs	307 (10.3%)	162 (10.4%)	145 (10.1%)	275 (10.9%)	32 (6.7%)
Missing	15 (0.5%)	10 (0.6%)	5 (0.3%)	10 (0.4%)	5 (1.0%)
Education levels, n (%)					
Elementary	449 (15.0%)	233 (14.9%)	216 (15.1%)	366 (14.5%)	83 (17.4%)
High school	1,485 (49.6%)	783 (50.1%)	702 (49.1%)	1,204 (47.9%)	281 (58.9%)
University and above	1,051 (35.1%)	545 (34.8%)	506 (35.4%)	940 (37.4%)	111 (23.3%)
Missing	8 (0.3%)	3 (0.2%)	5 (0.3%)	6 (0.2%)	2 (0.4%)
Partnered, n (%)					
No	1,543 (51.6%)	806 (51.5%)	737 (51.6%)	1,242 (49.4%)	301 (63.1%)
Yes	1,441 (48.1%)	754 (48.2%)	687 (48.1%)	1,267 (50.4%)	174 (36.5%)
Missing	9 (0.3%)	4 (0.3%)	5 (0.3%)	7 (0.3%)	2 (0.4%)
Physical activity, n (%)					
no or mild activity	2,004 (67.0%)	1,068 (68.3%)	936 (65.5%)	1,682 (66.9%)	322 (67.5%)
high activity	649 (21.7%)	311 (19.9%)	338 (23.7%)	585 (23.3%)	64 (13.4%)
Missing	340 (11.4%)	185 (11.8%)	155 (10.8%)	249 (9.9%)	91 (19.1%)
MMSE scores, mean ± SD	28.8 ± 1.4	28.7 ± 1.4	28.8 ± 1.4	28.9 ± 1.3	28.0 ± 1.7
BMI (kg/m^2^), mean ± SD	25.7 ± 4.1	25.6 ± 4.1	25.8 ± 4.1	25.8 ± 4.1	25.1 ± 4.3
No. of medications, mean ± SD	3.8 ± 3.3	3.9 ± 3.3	3.6 ± 3.2	3.7 ± 3.2	4.5 ± 3.5
APOE‐ε4 status, n (%)					
no ε4	1,950 (65.2%)	1,014 (64.8%)	936 (65.5%)	1,681 (66.8%)	269 (56.4%)
ε4	792 (26.5%)	414 (26.5%)	378 (26.5%)	628 (25.0%)	164 (34.4%)
Missing	251 (8.4%)	136 (8.7%)	115 (8.0%)	207 (8.2%)	44 (9.2%)
5‐Year average PM_2.5_ before baseline, n (%)					
< 8.4 µg/m^3^	1,809 (60.4%)	725 (46.4%)	1,084 (75.9%)	1,539 (61.2%)	270 (56.6%)
≥ 8.4 µg/m^3^	1,184 (39.6%)	839 (53.6%)	345 (24.1%)	977 (38.8%)	207 (43.4%)
5‐Year average water coverage (blue space access), n (%)					
No (= 0)	1,427 (47.7%)	749 (47.9%)	678 (47.4%)	1,175 (46.7%)	252 (52.8%)
Yes (> 0)	1,566 (52.3%)	815 (52.1%)	751 (52.6%)	1,341 (53.3%)	225 (47.2%)
5‐Year average road traffic noise before event, n (%)					
< 57.9 dB	1,380 (46.1%)	473 (30.2%)	907 (63.5%)	1,185 (47.1%)	195 (40.9%)
≥ 57.9 dB	1,613 (53.9%)	1,091 (69.8%)	522 (36.5%)	1,331 (52.9%)	282 (59.1%)

*Note*: Data are presented as mean ± standard deviation (SD) for continuous measures, and n (%) for categorical measures. The 5‐year average greenspace exposure prior to event was evaluated by NDVI and categorized based on the median as high (NDVI ≥ 0.28) and low (NDVI < 0.28).

Abbreviations: APOE, apolipoprotein E; BMI, body mass index; MMSE, Mini‐Mental State Examination; NDVI, Normalized Difference Vegetation Index; PM, particulate matter; SD, standard deviation; SES, socioeconomic status.

Results on the association between greenspace exposure and dementia are shown in Table 2. In the fully adjusted main model, per IQR higher greenspace exposure in a 100‐m buffer over the 5 years prior to dementia was associated with around 20% decreased dementia hazard (HR 0.79, 95% CI: 0.64, 0.96). A similar effect, although attenuated, was found for the 5‐ to 10‐year exposure window, whereby per IQR increment in greenspace was associated with a 14% decreased hazard of dementia (HR 0.86, 95% CI: 0.70, 1.06). The protective effects of greenspace exposure were attenuated for larger buffer zones (Table 2).

**TABLE 2:** HR with 95% CIs of dementia incidence by residential greenspace at different buffer zones and time windows.

Exposure windows	Model 1 HR (95%CI)	Model 2 HR (95%CI)	Model 3 HR (95%CI)
Greenspace 100 m buffer			
5‐Year mean NDVI	0.86 (0.75, 1.00)	0.82 (0.69, 0.97)	0.79 (0.64, 0.96)
10‐ to 5‐Year mean NDVI	0.95 (0.81, 1.10)	0.87 (0.73, 1.05)	0.86 (0.70, 1.06)
Greenspace 250 m buffer			
5‐Year mean NDVI	0.93 (0.80, 1.09)	0.89 (0.74, 1.06)	0.88 (0.71, 1.09)
10‐ to 5‐Year mean NDVI	1.06 (0.90, 1.24)	0.97 (0.80, 1.19)	0.99 (0.80, 1.24)
Greenspace 500 m buffer			
5‐Year mean NDVI	1.00 (0.86, 1.16)	0.93 (0.78, 1.12)	0.94 (0.76, 1.15)
10‐ to 5‐Year mean NDVI	1.10 (0.95, 1.28)	1.01 (0.84, 1.23)	1.04 (0.84, 1.28)

*Note*: Hazard ratios expressed per interquartile range increase in green space (IQR = 0.18). Model 1 adjusted for age and sex. Model 2 adjusted for model 1 + SES, education, smoking, physical activity, early retirement, civil status, number of medications, BMI, and neighborhood household mean income. Model 3 adjusted for model 2 + 5‐year average levels of PM_2.5_ before baseline, 5‐year average water coverage (blue space) at 100 m buffer before event and 5‐year road traffic noise before event.

Abbreviations: BMI, body mass index; CI, confidence interval; HR, hazard ratio; NDVI, Normalized Difference Vegetation Index; SES, socioeconomic status.

The analyses by potential modifiers are shown in Figure 1. We did not observe statistically significant differences in the associations between greenspace exposure and incidence of dementia depending on age, PM_2.5_ exposure, blue space access, and noise exposure (all *p*‐values for these interactions > 0.1). In contrast, we observed a stronger protective effect of greenspace exposure on dementia hazard among female participants (HR 0.69, 95% CI: 0.54, 0.89; *P_interaction_* = 0.082), than among male participants (HR 0.93, 95% CI: 0.71, 1.24). We observed a marginal interaction between greenspace and *APOE* ε4 status, whereby a protective effect of greenspace was observed among *APOE* ε4 non‐carriers (HR 0.72, 95% CI: 0.56 to 0.92; *p_interaction_* = 0.183), but not among *APOE* ε4 carriers (HR 0.91, 95% CI: 0.67 to 1.22).

**FIGURE 1:**
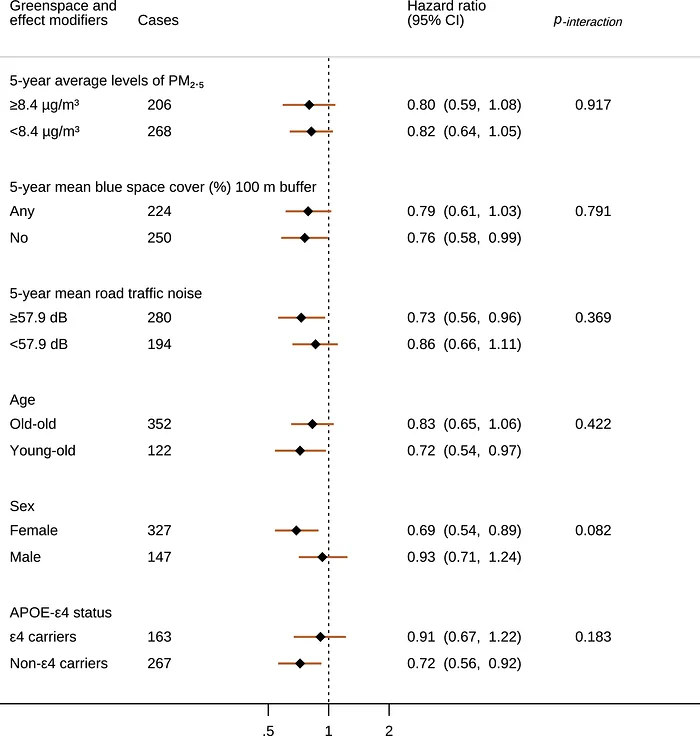
The effect of residential greenspace on dementia by levels of effect modifiers. Hazard ratio (HR) per interquartile range (IQR: 0.18) greenspace, in terms of 5‐year average levels of Normalized Difference Vegetation Index (NDVI) within a 100 m buffer zone before event, was derived from the Cox‐proportional hazard models. Models were adjusted for age and sex, socioeconomic status (SES), education, smoking, physical activity, early retirement, civil status, number of medications, body mass index (BMI), neighborhood household mean income, 5‐year average levels of particulate matter (PM2.5) air pollution before baseline, 5‐year average water coverage (blue space) at 100 m buffer before event and 5‐year average levels of road traffic noise before event.

### 3.2 Sensitivity analyses

The results remained similar to the main results when excluding the participants who changed their addresses during the follow‐up (Table S2 and Figure S1). We also observed similar results considering the dropout using IPW in the analyses (Table S3).

## 4 DISCUSSION

In this study, we investigated the association between residential greenspace, in the immediate individual surroundings (100‐m buffer) to the neighborhood level (500‐m buffer), and dementia incidence in a Swedish cohort of older adults followed up during 16 years. We found that one IQR increase in greenspace in the immediate residential surrounding (100 m) was associated with approximately a 20% reduced hazard of dementia, while controling for individual‐level and contextual factors, including air pollution (PM_2.5_) and road traffic noise exposure. In line with some previous findings, we observed stronger protective effects of greenspace on dementia incidence among women than men,^12^ and among *APOE*‐ε4 non‐carriers than carriers.^33^ The latter suggests that residential greenspace may be more strongly associated with lower dementia risk among individuals without genetic predisposition to dementia. However, as the interaction by *APOE*‐ε4 status was not statistically significant (*p* = 0.183), this should be interpreted cautiously. We did not observe significant interactions between greenspace and other residential environment exposures on dementia risk.

Importantly, there is yet a scarcity of studies on greenspace and dementia that have assessed greenspace in individuals’ immediate residential surroundings, that is, with high spatial precision,^12^, ^14^, ^15^ while also having a long follow‐up time, thorough in‐person clinical assessment of dementia, and including other important individual and environmental factors, as in our present study. This study thus contributes with novel knowledge and additional nuances to prior research by elucidating the potential significance of greenspace in the immediate residential surroundings in contributing to reducing dementia risk and identifying which factors modify this association.

### 4.1 Previous evidence

While some prior research has found residential greenspace to be a protective factor for cognitive aging and dementia, findings have been mixed. Most prior studies have measured residential greenspace at an aggregated level, for example, using post code areas, Lower Layer Super Output Areas (LSOA)^1^, larger areas or buffers of ≥500 m.^12^, ^14^, ^15^ Several studies found greenspace associated with reduced hazards of dementia as assessed via administrative health registers, including Brown et al. (2018) assessing greenspace within census blocks among Miami‐Dade Medicare beneficiaries, U.S.^34^; Klompmaker et al. (2022) studying greenspace in zip‐code areas of U.S. Medicare beneficiaries^35^; Paul et al. (2020) assessing greenspace within 250 m buffers of postcode centroids in register cohorts in Ontario, Canada^36^; Rodriguez‐Loureiro et al. (2022) finding greenspace within 500 m buffers associated with decreased AD, vascular and unspecified dementia mortality among urban residents in Belgium^37^; and Hu et al. (2023), studying greenspace cover within 300 and 1000 m of residential LSOAs in the UK Biobank.^38^ Wu & Jackson (2021) found greenspace land cover associated with lower dementia risk at an aggregated zip‐code area level in Mid‐Atlantic United States.^39^ Slawsky et al. (2022) found greenspace within a 2 km buffer associated with lower incident all‐cause dementia determined via psychiatric follow‐up assessments in 3049 older adults across four U.S. sites.^40^ Residential greenspace measured at an aggregated or neighbourhood scale has also been found to be associated with better cognitive functioning and less decline assessed via cognitive testing.^33^, ^41^, ^42^, ^43^

However, several studies reported mixed results. Astell‐Burt et al. (2020) found greenspace indicators within 1.6 km network buffers of residential block centroids associated with decreased or increased hazard of AD depending on the type of register‐based dementia indicator in a large Australian sample.^44^ Godina et al. (2023) investigated overall greenspace, forest land cover and greenspace diversity within 5 km buffers and found no association for overall greenspace, while forest cover was associated with lower MCI risk only, and greenspace diversity with lower dementia risk only.^45^ Yuchi et al. (2020) found greenspace within a 100 m buffer of post‐code centroids associated with reduced hazard of non‐AD dementia but increased AD risk in Metro Vancouver cohorts.^46^ Wu et al. (2017) investigated greenspace within a 100 m buffer of post‐code centroids and observed a positive association with cognitive status only in a sub‐sample living in high‐conurbation areas, while a negative association with cognitive function and no association with dementia was found for the whole sample of older adults across three cities in England.^47^ In a similar prior study, greenspace was associated with an increased hazard of cognitive impairment and dementia.^48^

Finally, some studies reported no association with dementia, including a study of greenspace within 300 and 1000 m buffers and cause‐specific mortality records in the Dutch longitudinal mortality database,^49^ a case–control study in Taiwan assessing greenspace at township level and dementia incidence via health claims records,^50^ and a UK multicenter cohort study assessing greenspace within 300 m network buffers of post‐code areas and dementia incidence.^51^

The variable findings across prior studies may reflect methodological constraints and heterogeneity in measures, samples, and contexts. Greenspace measures have generally been constrained to more crude, area‐level indicators or large buffer zones,^12^, ^14^, ^15^ increasing the risk of exposure misclassification^2^ and limiting evaluations of individuals’ immediate residential surroundings.^5^, ^14^ Furthermore, dementia has in most prior studies been assessed via register‐based data, which may vary between countries and samples regarding how well dementia is captured.^26^, ^52^

### 4.2 Possible pathways and future studies

Residential greenspace may support brain health and cognitive preservation via several pathways. Greenspace may exert benefits on different aspects of mental health^5^, ^6^, ^7^ and cardiometabolic health,^5^, ^8^ which in turn affect dementia risk.^2^, ^11^, ^12^, ^14^ Specific observed effects of greenspace exposure include improved stress mitigation,^18^, ^19^, ^20^ affect,^16^ cognitive performance,^17^ and facilitation of health‐related behaviors including better sleep,^4^ physical activity, and social stimulation and cohesion.^9^, ^12^, ^14^, ^22^ Stress may influence physiological inflammatory processes and atherosclerotic progression contributing to the development of cardiometabolic and cerebral vascular diseases, which are heavily linked to cognitive decline and progression to dementia.^2^, ^11^ Prior research has also indicated that the immediate/momentary positive effects of nature interactions on cognitive performance seem to be stronger among more cognitively vulnerable individuals and seem to be especially pertinent to executive cognitive processes.^17^ These in turn are important for the regulation of behavior, emotions, stress responses, and for effortful learning and memory,^10^ another resilience factor against cognitive decline and dementia.^2^

Greenspace may also support health indirectly by contributing to protection against hazardous environmental exposures like traffic noise, air pollution, and extreme heat.^23^, ^24^, ^53^ Air pollution and traffic noise have been associated with adverse cardiovascular and cognitive health outcomes.^25^, ^26^, ^27^ Extreme heat exposure is associated with increased cardiorespiratory morbidity and premature mortality.^53^ These direct and indirect health‐supporting functions are additional to the broader ecosystem services greenspace can provide, including climate change mitigation and biodiversity conservation.^24^ With continuous urbanization and densification,^24^ the risks increase of greenspace deprivation, as well as exposure to other urban environmental hazards like air pollution, noise, and extreme heat,^24^ highlighting the public health relevance of greenspace protection and regeneration.

Future studies should further investigate the role of individual‐level greenspace exposures on neurodegenerative biomarkers, cognitive decline and dementia longitudinally, and delineate the pathways of action, including psychobiological, behavioral, and social aspects, as well as possible mitigation effects of other hazardous environmental exposures, and intermediary mental and cardiometabolic health risk conditions.^13^, ^15^

### 4.3 Strengths and limitations

The present study has several strengths. Firstly, the study had a long follow‐up time (up to 16 years) and dementia was assessed via extensive in‐person assessments carried out regularly for the diagnosis of dementia and complemented with data from the Swedish Death Register and clinical records, enabling high validity in the detection of incident dementia cases. Secondly, residential greenness was assessed longitudinally with high precision, from the immediate surrounding of the home (100 m) that people are more automatically exposed to, to the neighborhood level (500 m), based on individual residential addresses, addressing a gap in prior studies of residential greenspace and dementia. Finally, important individual risk factors and other environmental exposures were considered in the analyses, including air pollution, traffic noise, blue space, neighborhood SES, demographic factors, and genetic risk.

There are also some limitations to consider. The sample was limited to a generally affluent urban area in Stockholm. Thus, observed dementia risk reduction estimates associated with residential greenness in this study may be generalizable primarily to such high‐urbanicity contexts. Next, there is potential selection bias due to attrition during follow‐ups in longitudinal studies. However, we found that dropouts had similar environmental exposures compared to remaining participants, and the results remained similar after including IPW in the analyses, suggesting that the risk of selection bias due to dropouts biasing the results is low. Finally, while the simultaneous consideration of multiple potentially protective and adverse environmental exposures is a strength, the study primarily assesses residential greenspace at different scales in relation to incident dementia, while controling for other exposures and testing whether greenspace levels interact with a set of other key residential environmental exposures and individual factors. Thus, a limitation of the study is that complexities in interactions between different exposures and their compound effects, beyond those investigated, are outside the scope of the present study. Future studies are warranted to shed further light on how compound exposome profiles contribute to shaping cognitive aging.

## 5 CONCLUSIONS

The results of this study suggest that residential greenspace, especially in the close surroundings of the home, is associated with a decreased hazard of dementia among older adults, while adjusting for other environmental exposures and individual factors. Moderation analyses showed that the protective effect of greenspace was present specifically among women and was stronger among non‐carriers of *APOE*‐ε4. Our findings provide empirical support for policy makers to preserve and improve urban greenspace in the immediate surroundings of residences as a potential resource for brain health and cognitive resilience in aging populations, in addition to its broader ecosystem services, environmental and public health benefits.^24^, ^54^, ^55^, ^56^

## AUTHOR CONTRIBUTIONS

C.S.: conceptualization, methodology, investigation, literature review, project management, writing—writing the original draft, writing—review and editing, funding acquisition, supervision. J.W.: data curation, formal analysis, writing—contribution to original draft, review and editing. A.P.: formal analysis, writing—review and editing. G.G.: data collection, writing—review and editing. ML: methodology, writing—review and editing. G.P.: methodology, writing—review and editing. D.R.: methodology, project management, data curation, writing—review and editing, funding acquisition, supervision. All the authors revised the manuscript for important intellectual content and approved the final version.

## CONFLICT OF INTEREST STATEMENT

The authors declare that they have no conflicts of interest. Author disclosures are available in the Supporting Information.

## CONSENT STATEMENT

Written informed consent was obtained from all participants or, if the person was severely cognitively impaired, from next of kin.

## Supporting information



Supporting Information

Supporting Information

Supporting Information

## Footnotes

1 Lower Layer Super Output Areas are consistent geographic units used in statistical analysis by the Office for National Statistics in the United Kingdom, https://www.ons.gov.uk

2 Also referred to as the Modifiable Areal Unit Problem, describing that when exposure is assessed using larger, aggregated spatial units, the measure becomes a less precise and more uncertain representation of an individual's true local exposure.
