# Supplementary material for: Precision residential greenspace, urban environmental exposures, and incident dementia among older adults in Sweden: A 16‐year cohort study
Source: Alzheimers Dement. 2026 Sep 28;22(10):e71835. doi: 10.1002/alz.71835 (PMC13619732; doi:10.1002/alz.71835)
Supplement: Supplementary file 1 [file ALZ-22-e71835-s002.pdf]

ICMJE DISCLOSURE FORM

**Date:**	7/7/2026
**Your Name:**	Cecilia U. D. Stenfors (CUDS)
**Manuscript Title:**	Precision residential greenspace, urban environmental exposures and incident dementia among older adults in Sweden: a 16-year cohort study
**Manuscript Number (if known):**	ADJ-D-26-01422

In the interest of transparency, we ask you to disclose all relationships/activities/interests listed below that are related to the content of your manuscript. "Related" means any relation with for-profit or not-for-profit third parties whose interests may be affected by the content of the manuscript. Disclosure represents a commitment to transparency and does not necessarily indicate a bias. If you are in doubt about whether to list a relationship/activity/interest, it is preferable that you do so.

The author's relationships/activities/interests should be defined broadly. For example, if your manuscript pertains to the epidemiology of hypertension, you should declare all relationships with manufacturers of antihypertensive medication, even if that medication is not mentioned in the manuscript.

In item #1 below, report all support for the work reported in this manuscript without time limit. For all other items, the time frame for disclosure is the past 36 months.

		**Name all entities with whom you have this relationship or indicate none (add rows as needed)**	**Specifications/Comments (e.g., if payments were made to you or to your institution)**
		Time frame: Since the initial planning of the work	
1	All support for the present manuscript (e.g., funding, provision of study materials, medical writing, article processing charges, etc.) No time limit for this item.	☐ None	
		Data collection of the Swedish National study on Aging and Care (SNAC-K) was supported by the Swedish Ministry of Health and Social Affairs; the participating County Councils and Municipalities; and the Swedish Research Council. This research was supported by grants from the Swedish Research Council for Health, Working Life and Welfare awarded to CUDS (Dnr 2020-00977) and DR (Dnr 2017-01768), and Region Stockholm awarded to CUDS (RS 2020-0501). J.W. was also supported by a PhD scholarship from the Chinese Scholarship Council (CSC, No. 201907930017), and a research grant from Lindhés Advokatbyrå AB (Nr: LA2020-0026). The funders had no role in study design, data collection, analysis and interpretation, the decision to publish, or preparation of the manuscript.	
			Click the tab key to add additional rows.
		Time frame: past 36 months	

		Name all entities with whom you have this relationship or indicate none (add rows as needed)	Specifications/Comments (e.g., if payments were made to you or to your institution)
**2**	Grants or contracts from any entity (*if not indicated in item #1 above*).	☒ **None**	
		None related to the present study/article, beyond reported research funding.	
**3**	Royalties or licenses	☒ **None**	
**4**	Consulting fees	☒ **None**	
		None related to the present study/article.	
**5**	Payment or honoraria for lectures, presentations, speakers bureaus, manuscript writing or educational events	☒ **None**	
**6**	Payment for expert testimony	☒ **None**	
**7**	Support for attending meetings and/or travel	☒ **None**	
		None beyond reported research funding.	
**8**	Patents planned, issued or pending	☒ **None**	
**9**	Participation on a Data Safety	☒ **None**	

		Name all entities with whom you have this relationship or indicate none (add rows as needed)	Specifications/Comments (e.g., if payments were made to you or to your institution)
	Monitoring Board or Advisory Board	None related to the present study/article.	
**10**	Leadership or fiduciary role in other board, society, committee or advocacy group, paid or unpaid	☒ **None**	
**11**	Stock or stock options	☒ **None**	
**12**	Receipt of equipment, materials, drugs, medical writing, gifts or other services	☒ **None**	
**13**	Other financial or non-financial interests	☒ **None**	

Please place an “X” next to the following statement to indicate your agreement:

☒ I certify that I have answered every question and have not altered the wording of any of the questions on this form.

ICMJE DISCLOSURE FORM

**Date:**	7/13/2026
**Your Name:**	Jing Wu (JW)
**Manuscript Title:**	Precision residential greenspace, urban environmental exposures and incident dementia among older adults in Sweden: a 16-year cohort study
**Manuscript Number (if known):**	ADJ-D-26-01422

In the interest of transparency, we ask you to disclose all relationships/activities/interests listed below that are related to the content of your manuscript. "Related" means any relation with for-profit or not-for-profit third parties whose interests may be affected by the content of the manuscript. Disclosure represents a commitment to transparency and does not necessarily indicate a bias. If you are in doubt about whether to list a relationship/activity/interest, it is preferable that you do so.

The author's relationships/activities/interests should be defined broadly. For example, if your manuscript pertains to the epidemiology of hypertension, you should declare all relationships with manufacturers of antihypertensive medication, even if that medication is not mentioned in the manuscript.

In item #1 below, report all support for the work reported in this manuscript without time limit. For all other items, the time frame for disclosure is the past 36 months.

		**Name all entities with whom you have this relationship or indicate none (add rows as needed)**	**Specifications/Comments (e.g., if payments were made to you or to your institution)**
		Time frame: Since the initial planning of the work	
1	All support for the present manuscript (e.g., funding, provision of study materials, medical writing, article processing charges, etc.) No time limit for this item.	☐ None	
		Data collection of the Swedish National study on Aging and Care (SNAC-K) was supported by the Swedish Ministry of Health and Social Affairs; the participating County Councils and Municipalities; and the Swedish Research Council.<br>This research was supported by grants from the Swedish Research Council for Health, Working Life and Welfare awarded to CUDS (Dnr 2020-00977) and DR (Dnr 2017-01768), and Region Stockholm awarded to CUDS (RS 2020-0501). J.W. was also supported by a PhD scholarship from the Chinese Scholarship Council (CSC, No. 201907930017), and a research grant from Lindhés Advokatbyrå AB (Nr: LA2020-0026).<br>The funders had no role in study design, data collection, analysis and interpretation, the decision to publish, or preparation of the manuscript.	
			Click the tab key to add additional rows.
		Time frame: past 36 months	

		Name all entities with whom you have this relationship or indicate none (add rows as needed)	Specifications/Comments (e.g., if payments were made to you or to your institution)
**2**	Grants or contracts from any entity (*if not indicated in item #1 above*).	☒ **None**	
		None related to the present study/article, beyond reported research funding.	
**3**	Royalties or licenses	☒ **None**	
**4**	Consulting fees	☒ **None**	
		None related to the present study/article.	
**5**	Payment or honoraria for lectures, presentations, speakers bureaus, manuscript writing or educational events	☒ **None**	
**6**	Payment for expert testimony	☒ **None**	
**7**	Support for attending meetings and/or travel	☒ **None**	
		None beyond reported research funding.	
**8**	Patents planned, issued or pending	☒ **None**	
**9**	Participation on a Data Safety	☒ **None**	

		Name all entities with whom you have this relationship or indicate none (add rows as needed)	Specifications/Comments (e.g., if payments were made to you or to your institution)
	Monitoring Board or Advisory Board	None related to the present study/article.	
**10**	Leadership or fiduciary role in other board, society, committee or advocacy group, paid or unpaid	☒ **None**	
**11**	Stock or stock options	☒ **None**	
**12**	Receipt of equipment, materials, drugs, medical writing, gifts or other services	☒ **None**	
**13**	Other financial or non-financial interests	☒ **None**	

Please place an "X" next to the following statement to indicate your agreement:

☒ I certify that I have answered every question and have not altered the wording of any of the questions on this form.

ICMJE DISCLOSURE FORM

**Date:**	7/14/2026
**Your Name:**	Giulia Grande (GG)
**Manuscript Title:**	Precision residential greenspace, urban environmental exposures and incident dementia among older adults in Sweden: a 16-year cohort study
**Manuscript Number (if known):**	ADJ-D-26-01422

In the interest of transparency, we ask you to disclose all relationships/activities/interests listed below that are related to the content of your manuscript. "Related" means any relation with for-profit or not-for-profit third parties whose interests may be affected by the content of the manuscript. Disclosure represents a commitment to transparency and does not necessarily indicate a bias. If you are in doubt about whether to list a relationship/activity/interest, it is preferable that you do so.

The author's relationships/activities/interests should be defined broadly. For example, if your manuscript pertains to the epidemiology of hypertension, you should declare all relationships with manufacturers of antihypertensive medication, even if that medication is not mentioned in the manuscript.

In item #1 below, report all support for the work reported in this manuscript without time limit. For all other items, the time frame for disclosure is the past 36 months.

		**Name all entities with whom you have this relationship or indicate none (add rows as needed)**	**Specifications/Comments (e.g., if payments were made to you or to your institution)**
		Time frame: Since the initial planning of the work	
1	All support for the present manuscript (e.g., funding, provision of study materials, medical writing, article processing charges, etc.) No time limit for this item.	☐ None	
		Data collection of the Swedish National study on Aging and Care (SNAC-K) was supported by the Swedish Research Council (ongoing/current grant: 2021-00178); the Swedish Ministry of Health and Social Affairs; the participating County Councils and Municipalities.<br><br>GG also received financial support from the Karolinska Institutet Strategic Research Area in Neuroscience in 2025; the Karolinska Institutet Committee for Research in 2025; from Alzheimerfonden (project number AF-1032487); Foundation for Age Research at Karolinska Institutet (project number 2025-02095); Swedish Society of Medicine (Svenska Läkaresällskapet project number: SLS-1022848 and SLS-1022882).<br><br>The funders had no role in study design, data collection, analysis and interpretation, the decision to publish, or preparation of the manuscript.	
			Click the tab key to add additional rows.
		Time frame: past 36 months	

		Name all entities with whom you have this relationship or indicate none (add rows as needed)	Specifications/Comments (e.g., if payments were made to you or to your institution)
**2**	Grants or contracts from any entity (*if not indicated in item #1 above*).	☒ **None**	
		None related to the present study/article, beyond reported research funding.	
**3**	Royalties or licenses	☒ **None**	
**4**	Consulting fees	☒ **None**	
		None related to the present study/article.	
**5**	Payment or honoraria for lectures, presentations, speakers bureaus, manuscript writing or educational events	☒ **None**	
**6**	Payment for expert testimony	☒ **None**	
**7**	Support for attending meetings and/or travel	☒ **None**	
		None beyond reported research funding.	
**8**	Patents planned, issued or pending	☒ **None**	
**9**	Participation on a Data Safety	☒ **None**	

		Name all entities with whom you have this relationship or indicate none (add rows as needed)	Specifications/Comments (e.g., if payments were made to you or to your institution)
	Monitoring Board or Advisory Board	None related to the present study/article.	
10	Leadership or fiduciary role in other board, society, committee or advocacy group, paid or unpaid	☒ **None**	
11	Stock or stock options	☒ **None**	
12	Receipt of equipment, materials, drugs, medical writing, gifts or other services	☒ **None**	
13	Other financial or non-financial interests	☒ **None**	

Please place an "X" next to the following statement to indicate your agreement:

☒ I certify that I have answered every question and have not altered the wording of any of the questions on this form.

ICMJE DISCLOSURE FORM

Date: 7/16/2026

Your Name: Andrei Pyko (AP)

Manuscript Title: Precision residential greenspace, urban environmental exposures and incident dementia among older adults in Sweden: a 16-year cohort study

Manuscript Number (if known): ADJ-D-26-01422

In the interest of transparency, we ask you to disclose all relationships/activities/interests listed below that are related to the content of your manuscript. "Related" means any relation with for-profit or not-for-profit third parties whose interests may be affected by the content of the manuscript. Disclosure represents a commitment to transparency and does not necessarily indicate a bias. If you are in doubt about whether to list a relationship/activity/interest, it is preferable that you do so.

The author's relationships/activities/interests should be defined broadly. For example, if your manuscript pertains to the epidemiology of hypertension, you should declare all relationships with manufacturers of antihypertensive medication, even if that medication is not mentioned in the manuscript.

In item #1 below, report all support for the work reported in this manuscript without time limit. For all other items, the time frame for disclosure is the past 36 months.

		Name all entities with whom you have this relationship or indicate none (add rows as needed)	Specifications/Comments (e.g., if payments were made to you or to your institution)
		**Time frame: Since the initial planning of the work**	
1	All support for the present manuscript (e.g., funding, provision of study materials, medical writing, article processing charges, etc.) No time limit for this item.	☐ None	
		Data collection of the Swedish National study on Aging and Care (SNAC-K) was supported by the Swedish Ministry of Health and Social Affairs; the participating County Councils and Municipalities; and the Swedish Research Council. This research was supported by grants from the Swedish Research Council for Health, Working Life and Welfare awarded to CUDS (Dnr 2020-00977) and DR (Dnr 2017-01768), and Region Stockholm awarded to CUDS (RS 2020-0501). J.W. was also supported by a PhD scholarship from the Chinese Scholarship Council (CSC, No. 201907930017), and a research grant from Lindhés Advokatbyrå AB (Nr: LA2020-0026). The funders had no role in study design, data collection, analysis and interpretation, the decision to publish, or preparation of the manuscript.	
			Click the tab key to add additional rows.
		**Time frame: past 36 months**	

		Name all entities with whom you have this relationship or indicate none (add rows as needed)	Specifications/Comments (e.g., if payments were made to you or to your institution)
**2**	Grants or contracts from any entity (*if not indicated in item #1 above*).	☒ **None**	
		None related to the present study/article, beyond reported research funding.	
**3**	Royalties or licenses	☒ **None**	
**4**	Consulting fees	☒ **None**	
		None related to the present study/article.	
**5**	Payment or honoraria for lectures, presentations, speakers bureaus, manuscript writing or educational events	☒ **None**	
**6**	Payment for expert testimony	☒ **None**	
**7**	Support for attending meetings and/or travel	☒ **None**	
		None beyond reported research funding.	
**8**	Patents planned, issued or pending	☒ **None**	
**9**	Participation on a Data Safety	☒ **None**	

		Name all entities with whom you have this relationship or indicate none (add rows as needed)	Specifications/Comments (e.g., if payments were made to you or to your institution)
	Monitoring Board or Advisory Board	None related to the present study/article.	
**10**	Leadership or fiduciary role in other board, society, committee or advocacy group, paid or unpaid	☒ **None**	
**11**	Stock or stock options	☒ **None**	
**12**	Receipt of equipment, materials, drugs, medical writing, gifts or other services	☒ **None**	
**13**	Other financial or non-financial interests	☒ **None**	

Please place an "X" next to the following statement to indicate your agreement:

☒ I certify that I have answered every question and have not altered the wording of any of the questions on this form.

ICMJE DISCLOSURE FORM

Date: 7/16/2026

Your Name: Mare Lõhmus

Manuscript Title: Precision residential greenspace, urban environmental exposures and incident dementia among older adults in Sweden: a 16-year cohort study

Manuscript Number (if known): ADJ-D-26-01422

In the interest of transparency, we ask you to disclose all relationships/activities/interests listed below that are related to the content of your manuscript. "Related" means any relation with for-profit or not-for-profit third parties whose interests may be affected by the content of the manuscript. Disclosure represents a commitment to transparency and does not necessarily indicate a bias. If you are in doubt about whether to list a relationship/activity/interest, it is preferable that you do so.

The author's relationships/activities/interests should be defined broadly. For example, if your manuscript pertains to the epidemiology of hypertension, you should declare all relationships with manufacturers of antihypertensive medication, even if that medication is not mentioned in the manuscript.

In item #1 below, report all support for the work reported in this manuscript without time limit. For all other items, the time frame for disclosure is the past 36 months.

		Name all entities with whom you have this relationship or indicate none (add rows as needed)	Specifications/Comments (e.g., if payments were made to you or to your institution)
		Time frame: Since the initial planning of the work	
1	All support for the present manuscript (e.g., funding, provision of study materials, medical writing, article processing charges, etc.)<br>No time limit for this item.	☐ None	
		Data collection of the Swedish National study on Aging and Care (SNAC-K) was supported by the Swedish Ministry of Health and Social Affairs; the participating County Councils and Municipalities; and the Swedish Research Council.<br>This research was supported by grants from the Swedish Research Council for Health, Working Life and Welfare awarded to CUDS (Dnr 2020-00977) and DR (Dnr 2017-01768), and Region Stockholm awarded to CUDS (RS 2020-0501). J.W. was also supported by a PhD scholarship from the Chinese Scholarship Council (CSC, No. 201907930017), and a research grant from Lindhés Advokatbyrå AB (Nr: LA2020-0026).<br>The funders had no role in study design, data collection, analysis and interpretation, the decision to publish, or preparation of the manuscript.	
			Click the tab key to add additional rows.
		Time frame: past 36 months	

		Name all entities with whom you have this relationship or indicate none (add rows as needed)	Specifications/Comments (e.g., if payments were made to you or to your institution)
**2**	Grants or contracts from any entity (*if not indicated in item #1 above*).	☒ **None**	
		None related to the present study/article, beyond reported research funding.	
**3**	Royalties or licenses	☒ **None**	
**4**	Consulting fees	☒ **None**	
		None related to the present study/article.	
**5**	Payment or honoraria for lectures, presentations, speakers bureaus, manuscript writing or educational events	☒ **None**	
**6**	Payment for expert testimony	☒ **None**	
**7**	Support for attending meetings and/or travel	☒ **None**	
		None beyond reported research funding.	
**8**	Patents planned, issued or pending	☒ **None**	
**9**	Participation on a Data Safety	☒ **None**	

		Name all entities with whom you have this relationship or indicate none (add rows as needed)	Specifications/Comments (e.g., if payments were made to you or to your institution)
	Monitoring Board or Advisory Board	None related to the present study/article.	
**10**	Leadership or fiduciary role in other board, society, committee or advocacy group, paid or unpaid	☒ **None**	
**11**	Stock or stock options	☒ **None**	
**12**	Receipt of equipment, materials, drugs, medical writing, gifts or other services	☒ **None**	
**13**	Other financial or non-financial interests	☒ **None**	

Please place an "X" next to the following statement to indicate your agreement:

☒ I certify that I have answered every question and have not altered the wording of any of the questions on this form.

ICMJE DISCLOSURE FORM

Date: 7/14/2026

Your Name: Göran Pershagen

Manuscript Title: Precision residential greenspace, urban environmental exposures and incident dementia among older adults in Sweden: a 16-year cohort study

Manuscript Number (if known): ADJ-D-26-01422

In the interest of transparency, we ask you to disclose all relationships/activities/interests listed below that are related to the content of your manuscript. "Related" means any relation with for-profit or not-for-profit third parties whose interests may be affected by the content of the manuscript. Disclosure represents a commitment to transparency and does not necessarily indicate a bias. If you are in doubt about whether to list a relationship/activity/interest, it is preferable that you do so.

The author's relationships/activities/interests should be defined broadly. For example, if your manuscript pertains to the epidemiology of hypertension, you should declare all relationships with manufacturers of antihypertensive medication, even if that medication is not mentioned in the manuscript.

In item #1 below, report all support for the work reported in this manuscript without time limit. For all other items, the time frame for disclosure is the past 36 months.

		**Name all entities with whom you have this relationship or indicate none (add rows as needed)**	**Specifications/Comments (e.g., if payments were made to you or to your institution)**
**Time frame: Since the initial planning of the work**			
1	All support for the present manuscript (e.g., funding, provision of study materials, medical writing, article processing charges, etc.) No time limit for this item.	☐ None	
		Data collection of the Swedish National study on Aging and Care (SNAC) was supported by the Swedish Ministry of Health and Social Affairs; the participating County Councils and Municipalities; and the Swedish Research Council. This research was supported by grants from the Swedish Research Council for Health, Working Life and Welfare awarded to CUDS (Dnr 2020-00977) and DR (Dnr 2017-01768), and Region Stockholm awarded to CUDS (RS 2020-0501). J.W. was also supported by a PhD scholarship from the Chinese Scholarship Council (CSC, No. 201907930017), and a research grant from Lindhés Advokatbyrå AB (Nr: LA2020-0026). The funders had no role in study design, data collection, analysis and interpretation, the decision to publish, or preparation of the manuscript.	
			Click the tab key to add additional rows.
**Time frame: past 36 months**			

		Name all entities with whom you have this relationship or indicate none (add rows as needed)	Specifications/Comments (e.g., if payments were made to you or to your institution)
**2**	Grants or contracts from any entity (*if not indicated in item #1 above*).	☒ **None**	
		None related to the present study/article, beyond reported research funding.	
**3**	Royalties or licenses	☒ **None**	
**4**	Consulting fees	☒ **None**	
		None related to the present study/article.	
**5**	Payment or honoraria for lectures, presentations, speakers bureaus, manuscript writing or educational events	☒ **None**	
**6**	Payment for expert testimony	☒ **None**	
**7**	Support for attending meetings and/or travel	☒ **None**	
		None beyond reported research funding.	
**8**	Patents planned, issued or pending	☒ **None**	
**9**	Participation on a Data Safety	☒ **None**	

		Name all entities with whom you have this relationship or indicate none (add rows as needed)	Specifications/Comments (e.g., if payments were made to you or to your institution)
	Monitoring Board or Advisory Board	None related to the present study/article.	
**10**	Leadership or fiduciary role in other board, society, committee or advocacy group, paid or unpaid	☒ **None**	
**11**	Stock or stock options	☒ **None**	
**12**	Receipt of equipment, materials, drugs, medical writing, gifts or other services	☒ **None**	
**13**	Other financial or non-financial interests	☒ **None**	

Please place an "X" next to the following statement to indicate your agreement:

☒ I certify that I have answered every question and have not altered the wording of any of the questions on this form.

ICMJE DISCLOSURE FORM

**Date:**	7/13/2026
**Your Name:**	Debora Rizzuto (DR)
**Manuscript Title:**	Precision residential greenspace, urban environmental exposures and incident dementia among older adults in Sweden: a 16-year cohort study
**Manuscript Number (if known):**	ADJ-D-26-01422

In the interest of transparency, we ask you to disclose all relationships/activities/interests listed below that are related to the content of your manuscript. "Related" means any relation with for-profit or not-for-profit third parties whose interests may be affected by the content of the manuscript. Disclosure represents a commitment to transparency and does not necessarily indicate a bias. If you are in doubt about whether to list a relationship/activity/interest, it is preferable that you do so.

The author's relationships/activities/interests should be defined broadly. For example, if your manuscript pertains to the epidemiology of hypertension, you should declare all relationships with manufacturers of antihypertensive medication, even if that medication is not mentioned in the manuscript.

In item #1 below, report all support for the work reported in this manuscript without time limit. For all other items, the time frame for disclosure is the past 36 months.

		Name all entities with whom you have this relationship or indicate none (add rows as needed)	Specifications/Comments (e.g., if payments were made to you or to your institution)
Time frame: Since the initial planning of the work			
1	All support for the present manuscript (e.g., funding, provision of study materials, medical writing, article processing charges, etc.) No time limit for this item.	☐ None	
		Data collection of the Swedish National study on Aging and Care (SNAC-K) was supported by the Swedish Ministry of Health and Social Affairs; the participating County Councils and Municipalities; and the Swedish Research Council (current grant: 2021-00178). This research was supported by grants from the Swedish Research Council for Health, Working Life and Welfare awarded to CUDS (Dnr 2020-00977) and DR (Dnr 2017-01768), and Region Stockholm awarded to CUDS (RS 2020-0501). J.W. was also supported by a PhD scholarship from the Chinese Scholarship Council (CSC, No. 201907930017), and a research grant from Lindhés Advokatbyrå AB (Nr: LA2020-0026). A grant from the Swedish Research Council for Sustainable development (FORMAS) was also awarded to DR (Dnr: 2025-02558). The funders had no role in study design, data collection, analysis and interpretation, the decision to publish, or preparation of the manuscript.	
			Click the tab key to add additional rows.
Time frame: past 36 months			

		Name all entities with whom you have this relationship or indicate none (add rows as needed)	Specifications/Comments (e.g., if payments were made to you or to your institution)
**2**	Grants or contracts from any entity (*if not indicated in item #1 above*).	☒ **None**	
**3**	Royalties or licenses	☒ **None**	
**4**	Consulting fees	☒ **None**	
**5**	Payment or honoraria for lectures, presentations, speakers bureaus, manuscript writing or educational events	☒ **None**	
**6**	Payment for expert testimony	☒ **None**	
**7**	Support for attending meetings and/or travel	☒ **None**	
		None beyond reported research funding.	
**8**	Patents planned, issued or pending	☒ **None**	
**9**	Participation on a Data Safety	☒ **None**	

		Name all entities with whom you have this relationship or indicate none (add rows as needed)	Specifications/Comments (e.g., if payments were made to you or to your institution)
	Monitoring Board or Advisory Board		
**10**	Leadership or fiduciary role in other board, society, committee or advocacy group, paid or unpaid	☒ **None**	
**11**	Stock or stock options	☒ **None**	
**12**	Receipt of equipment, materials, drugs, medical writing, gifts or other services	☒ **None**	
**13**	Other financial or non-financial interests	☒ **None**	

Please place an "X" next to the following statement to indicate your agreement:

☒ I certify that I have answered every question and have not altered the wording of any of the questions on this form.
