# Supplementary material for: Precision residential greenspace, urban environmental exposures, and incident dementia among older adults in Sweden: A 16‐year cohort study
Source: Alzheimers Dement. 2026 Sep 28;22(10):e71835. doi: 10.1002/alz.71835 (PMC13619732; doi:10.1002/alz.71835)

Greenspace and effect modifiers	Cases	Hazard ratio (95% CI)	*p*-interaction
5-year average levels of $PM_{2.5}$			
≥8.4 µg/m³	200	0.77 (0.56, 1.05)	0.877
<8.4 µg/m³	255	0.79 (0.61, 1.02)	
5-year mean blue space cover (%) 100 m buffer			
Any	217	0.78 (0.60, 1.02)	0.614
No	238	0.71 (0.54, 0.95)	
5-year mean road traffic noise			
≥57.9 dB	274	0.73 (0.55, 0.95)	0.434
<57.9 dB	181	0.84 (0.64, 1.10)	
Age			
Old-old	345	0.79 (0.62, 1.01)	0.558
Young-old	110	0.71 (0.51, 0.98)	
Sex			
Female	317	0.67 (0.52, 0.87)	0.101
Male	138	0.90 (0.67, 1.21)	
APOE-ε4 status			
ε4 carriers	152	0.88 (0.64, 1.21)	0.210
Non-ε4 carriers	259	0.70 (0.54, 0.90)	

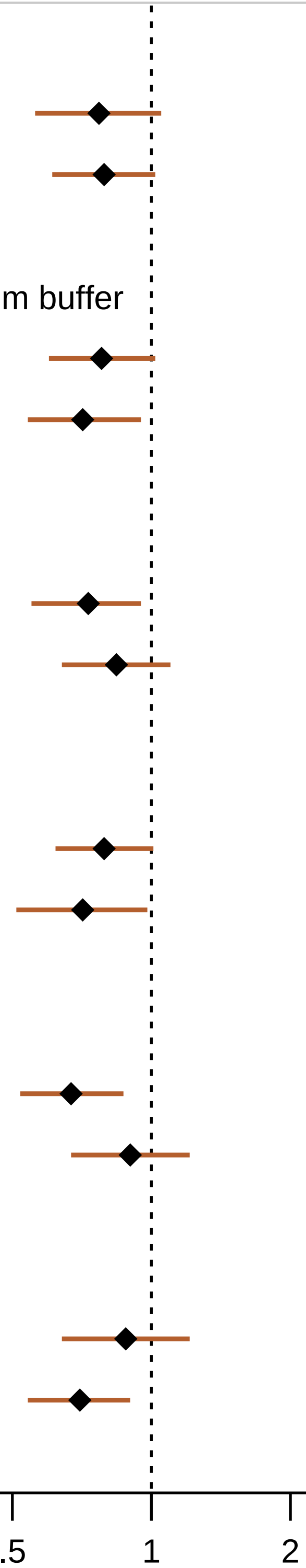

Supplement: Supplementary file 2 [file ALZ-22-e71835-s003.pdf]
