# Supplementary material for: Precision residential greenspace, urban environmental exposures, and incident dementia among older adults in Sweden: A 16‐year cohort study
Source: Alzheimers Dement. 2026 Sep 28;22(10):e71835. doi: 10.1002/alz.71835 (PMC13619732; doi:10.1002/alz.71835)
Supplement: Supplementary file 3 [file ALZ-22-e71835-s001.docx]

## **Supplementary information**

**Precision residential greenspace, urban environmental exposures and incident dementia among older adults in Sweden: a 16-year cohort study**

Cecilia U. D. Stenfors^a,b,e^*, Jing Wu^b,e^, Giulia Grande^b^, Andrei Pyko^c,d^, Mare Löhmus^c,d^, Göran Pershagen^c^, and Debora Rizzuto^b^

a. Department of Psychology, Stockholm University, Stockholm, Sweden.
b. Aging Research Center, Department of Neurobiology, Care Sciences and Society, Karolinska Institute, Stockholm, Sweden.
c. Institute of Environmental Medicine, Karolinska Institute, Stockholm, Sweden.
d. Center for Occupational and Environmental Medicine, Region Stockholm, Stockholm, Sweden.

^e^ These authors share the first authorship.

***Corresponding author:**

Cecilia U. D. Stenfors, [cecilia.stenfors@psychology.su.se](mailto:cecilia.stenfors@psychology.su.se)

Department of Psychology, Stockholm University

Albanovägen 12, 106 91 Stockholm, Sweden

# Table S1. Baseline characteristics of the overall sample and dropout.

|  | **Total** | **Not drop-out** | **Drop-out** | **p-value** |
| --- | --- | --- | --- | --- |
|  | **N=2,993** | **N=2,494** | **N=499** |  |
| Age at baseline, mean±SD | 73.1±10.4 | 72.9±10.5 | 74.1±9.7 | 0.022 |
| Sex, n (%) |  |  |  | 0.004 |
| Men | 1,118 (37.4%) | 960 (38.5%) | 158 (31.7%) |  |
| Women | 1,875 (62.6%) | 1,534 (61.5%) | 341 (68.3%) |  |
| SES status, n (%) |  |  |  | 0.31 |
| Blue collar workers | 650 (21.7%) | 532 (21.3%) | 118 (23.6%) |  |
| White collar workers | 2,021 (67.5%) | 1,699 (68.1%) | 322 (64.5%) |  |
| Entrepreneurs | 307 (10.3%) | 251 (10.1%) | 56 (11.2%) |  |
| Missing | 15 (0.5%) | 12 (0.5%) | 3 (0.6%) |  |
| Education levels, n (%) |  |  |  | 0.018 |
| Elementary | 449 (15.0%) | 359 (14.4%) | 90 (18.0%) |  |
| High school | 1,485 (49.6%) | 1,229 (49.3%) | 256 (51.3%) |  |
| University and above | 1,051 (35.1%) | 900 (36.1%) | 151 (30.3%) |  |
| Missing | 8 (0.3%) | 6 (0.2%) | 2 (0.4%) |  |
| Partnered, n (%) |  |  |  | 0.24 |
| No | 1,543 (51.6%) | 1,275 (51.1%) | 268 (53.7%) |  |
| Yes | 1,441 (48.1%) | 1,214 (48.7%) | 227 (45.5%) |  |
| Missing | 9 (0.3%) | 5 (0.2%) | 4 (0.8%) |  |
| Physical activity, n (%) |  |  |  | 0.006 |
| no or mild activity | 2,004 (67.0%) | 1,656 (66.4%) | 348 (69.7%) |  |
| high activity | 649 (21.7%) | 566 (22.7%) | 83 (16.6%) |  |
| Missing | 340 (11.4%) | 272 (10.9%) | 68 (13.6%) |  |
| MMSE scores, mean±SD | 28.8±1.4 | 28.8±1.4 | 28.6±1.5 | 0.017 |
| BMI (kg/m²), mean±SD | 25.7±4.1 | 25.6±4.1 | 25.8±4.3 | 0.35 |
| Number of medications, mean±SD | 3.8±3.3 | 3.8±3.3 | 3.8±3.2 | 0.93 |
| ApoE-4 status, n (%) |  |  |  | 0.32 |
| no E4 | 1,950 (65.2%) | 1,671 (67.0%) | 279 (55.9%) |  |
| E4 | 792 (26.5%) | 667 (26.7%) | 125 (25.1%) |  |
| Missing | 251 (8.4%) | 156 (6.3%) | 95 (19.0%) |  |
| 5-year average PM_2.5_ before baseline, n (%) |  |  |  | 0.97 |
| <8.4 µg/m3 | 1,809 (60.4%) | 1,507 (60.4%) | 302 (60.5%) |  |
| ≥8.4 µg/m3 | 1,184 (39.6%) | 987 (39.6%) | 197 (39.5%) |  |
| 5-year average water coverage (blue space access), n (%) |  |  |  | 0.17 |
| =0 | 1,428 (47.7%) | 1,176 (47.2%) | 252 (50.5%) |  |
| >0 | 1,565 (52.3%) | 1,318 (52.8%) | 247 (49.5%) |  |
| 5-year average road traffic noise before event, n (%) |  |  |  | 0.65 |
| < 57.9 dB | 1,383 (46.2%) | 1,157 (46.4%) | 226 (45.3%) |  |
| ≥ 57.9 dB | 1,610 (53.8%) | 1,337 (53.6%) | 273 (54.7%) |  |
| 5-year average greenspace (NDVI) before event, n (%) |  |  |  | 0.84 |
| Low greenspace (<0.28) | 1,565 (52.3%) | 1,302 (52.2%) | 263 (52.7%) |  |
| High greenspace (≥0.28) | 1,428 (47.7%) | 1,192 (47.8%) | 236 (47.3%) |  |

*Note, table S1:* Data are presented as mean±standard deviation (SD) for continuous measures, and n (%) for categorical measures. Abbreviations: BMI: body mass index; SES, socioeconomic status; SD, standard deviation; PM, particulate matter; NDVI, Normalized Difference Vegetation Index.

# Table S2. Hazard ratio (HR) with 95% confidence interval of incidence dementia by greenspace exposure at different buffers and time windows*, excluding participants who changed addresses during follow-ups* (n = 349).

| Exposure windows | Model 1 | Model 2 | Model 3 |
| --- | --- | --- | --- |
|  | HR (95%CI) | HR (95%CI) | HR (95%CI) |
| 100m buffer |  |  |  |
| 5-year mean NDVI | 0.86 (0.74, 1.00) | 0.81 (0.67, 0.97) | 0.76 (0.61, 0.94) |
| 10-5 year mean NDVI | 0.96 (0.82, 1.12) | 0.89 (0.73, 1.08) | 0.87 (0.70, 1.08) |
| 250m buffer |  |  |  |
| 5-year mean NDVI | 0.93 (0.79, 1.09) | 0.86 (0.71, 1.05) | 0.83 (0.66, 1.04) |
| 10-5 year mean NDVI | 1.08 (0.92, 1.27) | 1.00 (0.81, 1.23) | 1.02 (0.81, 1.28) |
| 500m buffer |  |  |  |
| 5-year mean NDVI | 0.99 (0.85, 1.17) | 0.91 (0.74, 1.12) | 0.89 (0.71, 1.12) |
| 10-5 year mean NDVI | 1.14 (0.98, 1.34) | 1.06 (0.86, 1.30) | 1.08 (0.86, 1.35) |

*Note, table S2:* Hazard ratios expressed per interquartile range increase in green space (IQR= 0.18). Model 1 adjusted for age and sex. Model 2 adjusted for model 1 + SES, education, smoking, physical activity, early retirement, civil status, number of medications, BMI, and neighborhood household mean income. Model 3 adjusted for model 2 + 5-year average levels of PM_2.5_ before baseline, 5-year average levels of water coverage at 100m buffer before event, and 5-year road traffic noise before event. Abbreviations: BMI, body mass index; CI, confidence interval; NDVI, Normalized Difference Vegetation Index; HR, hazard ratio.

# Figure S1. The effect of greenspace on dementia by levels of effect modifiers, *excluding participants who changed addresses during follow-ups* (n = 349)

*Note, figure S1:* HR per interquartile range (IQR: 0.18) was derived from the Cox-proportional hazard models. Models were adjusted for age and sex, SES, education, smoking, physical activity, early retirement, civil status, number of medications, BMI, neighborhood household mean income, 5-year average water coverage at 100m buffer before event, and 5-year road traffic noise before event.

# Table S3. Hazard ratio (HR) with 95% confidence interval of incidence dementia by greenspace exposure at different buffers and time windows *including inverse probability weight.*

| Exposure windows | Model 1 | Model 2 | Model 3 |
| --- | --- | --- | --- |
|  | HR (95%CI) | HR (95%CI) | HR (95%CI) |
| 100m buffer |  |  |  |
| 5-year mean NDVI | 0.86 (0.74, 0.99) | 0.81 (0.68, 0.96) | 0.78 (0.64, 0.96) |
| 10-5 year mean NDVI | 0.94 (0.81, 1.09) | 0.86 (0.72, 1.04) | 0.86 (0.70, 1.06) |
| 250m buffer |  |  |  |
| 5-year mean NDVI | 0.93 (0.80, 1.08) | 0.88 (0.74, 1.05) | 0.88 (0.72, 1.08) |
| 10-5 year mean NDVI | 1.05 (0.89, 1.23) | 0.97 (0.79, 1.17) | 0.99 (0.80, 1.24) |
| 500m buffer |  |  |  |
| 5-year mean NDVI | 0.99 (0.85, 1.15) | 0.93 (0.78, 1.11) | 0.94 (0.77, 1.14) |
| 10-5 year mean NDVI | 1.10 (0.95, 1.28) | 1.01 (0.84, 1.22) | 1.04 (0.85, 1.28) |

*Note, table S3:* Hazard ratios expressed per interquartile range increase in green space (IQR= 0.18). Model 1 adjusted for age and sex. Model 2 adjusted for model 1 + SES, education, smoking, physical activity, early retirement, civil status, number of medications, BMI, and neighborhood household mean income. Model 3 adjusted for model 2 + 5-year average levels of PM_2.5_ before baseline, 5-year average levels of water coverage at 100m buffer before event, and 5-year road traffic noise before event. Abbreviations: BMI, body mass index; CI, confidence interval; NDVI, Normalized Difference Vegetation Index; HR, hazard ratio.
